# Supplementary material for: Single Amino Acids as Sole Nitrogen Source for the Production of Lipids and Coenzyme Q by Thraustochytrium sp. RT2316-16
Source: Microorganisms. 2024 Jul 14;12(7):1428. doi: 10.3390/microorganisms12071428 (PMC11279195; doi:10.3390/microorganisms12071428)
Supplement: Supplementary file 1 [file microorganisms-12-01428-s001.zip › microorganisms-3079885-supplementary.pdf]

Supplemental Material

Single Amino Acids as Sole Nitrogen Source for the Production of Lipids and Coenzyme Q by *Thraustochytrium* sp. RT2316-16

Liset Flores and Carolina Shene

Table S1. Experimental data of the growth curves of *Thraustochytrium* sp. RT2316-16 in different media and kinetic parameters estimated from data.

	Time (h)	Biomass (g/L)	Xlf (g/L)	Glucose (g) (g/L)	Lipids (%)	Q_{10} (mg/gDW)	Q_9 (μg/gDW)	Lipids (g/g xlf)	L (g/L)	μ_{xlf} (h^{-1})	μ_{xt} (h^{-1})	r_g (g/h g DW)	r_L (g/h g DW)
Cysteine	0	0.31	0.24	5.00	22.57	4.71	62.50	0.29	0.07				
	12	0.35	0.33	4.78	7.08	167.41	3.50	0.08	0.02	0.025	0.009	0.055	-0.012
	24	1.25	1.14	4.40	8.47	22.89	12.50	0.09	0.11	0.105	0.106	0.040	0.116
	36	1.45	1.32	3.56	8.95	10.37	14.50	0.10	0.13	0.012	0.012	0.052	0.040
	48	1.73	1.62	1.76	6.09	11.81	17.25	0.06	0.10	0.017	0.014	0.094	-0.239
	60	1.90	1.78	0.23	6.31	8.76	19.00	0.07	0.12	0.008	0.008	0.070	0.018
	72	2.35	2.23	0.00	5.32	6.60	23.50	0.06	0.12	0.019	0.018	0.009	-0.082
	84	2.37	2.27	0.00	4.42	2.84	23.75	0.05	0.10	0.002	0.001	0.000	-0.075
	96	2.83	2.71	0.00	4.06	0.15	28.25	0.04	0.11	0.015	0.014	0.000	-0.030
Proline-1	0	0.31	0.24	5.00	22.57	4.71	62.50	0.29	0.07				
	12	0.60	0.55	4.90	9.16	20.75	6.00	0.10	0.05	0.068	0.054	0.019	-1.117
	24	1.57	1.44	4.29	8.55	11.03	15.75	0.09	0.13	0.081	0.080	0.047	-0.051
	36	1.62	1.49	3.60	8.31	16.83	16.25	0.09	0.14	0.003	0.003	0.036	-0.020
	48	1.83	1.68	3.37	7.68	2.48	18.25	0.08	0.14	0.010	0.010	0.011	-0.052
	60	2.17	2.02	3.25	7.15	7.22	21.75	0.08	0.16	0.015	0.015	0.005	-0.044
	72	2.62	2.53	2.91	3.62	0.05	26.25	0.04	0.09	0.019	0.016	0.012	-0.295
	84	2.80	2.70	3.14	3.75	0.02	28.00	0.04	0.10	0.005	0.005	-0.007	-0.011
	96	3.37	3.23	3.25	4.30	0.01	33.75	0.04	0.15	0.015	0.016	0.003	0.046
Lysine	0	0.31	0.24	5.00	22.57	4.71	62.50	0.29	0.07				
	12	1.38	1.23	4.63	10.54	2.13	13.75	0.12	0.14	0.135	0.123	0.037	-1.002
	24	1.40	1.24	4.32	11.44	1.60	14.00	0.13	0.16	0.001	0.002	0.018	0.075

	36	1.85	1.66	3.79	10.53	1.56	18.50	0.12	0.19	0.024	0.023	0.027	-0.076
	48	2.10	1.84	3.79	12.38	1.58	21.00	0.14	0.26	0.009	0.011	0.000	0.154
	60	2.17	1.98	3.06	8.74	0.00	21.75	0.10	0.19	0.006	0.003	0.028	-0.304
	72	2.57	2.36	2.45	8.54	0.00	25.75	0.09	0.22	0.014	0.014	0.021	-0.016
	84	2.82	2.66	0.96	5.67	0.02	28.25	0.06	0.16	0.010	0.008	0.046	-0.239
	96	3.20	3.06	0.75	4.53	0.01	32.00	0.05	0.14	0.011	0.010	0.006	-0.095
Glutamine	0	0.31	0.24	5.00	22.57	4.71	62.50	0.29	0.07				
	12	1.53	1.33	4.67	12.74	0.00	15.25	0.15	0.19	0.142	0.132	0.030	-0.819
	24	2.12	1.89	4.17	11.06	0.00	21.25	0.12	0.24	0.029	0.028	0.023	-0.140
	36	2.20	1.88	3.60	14.75	0.00	22.00	0.17	0.32		0.003	0.022	0.308
	48	2.65	2.17	1.95	18.30	0.06	26.50	0.22	0.48	0.012	0.016	0.057	0.295
	60	2.95	2.49	1.34	15.62	0.01	29.50	0.19	0.46	0.012	0.009	0.018	-0.223
	72	3.10	2.72	0.97	12.42	0.00	31.00	0.14	0.38	0.007	0.004	0.010	-0.266
	84	3.30	3.01	0.76	8.79	0.07	33.00	0.10	0.29	0.009	0.005	0.005	-0.303
	96	3.55	3.36	0.76	5.35	0.05	35.50	0.06	0.19	0.009	0.006	0.000	-0.286
Proline-2	0	0.30	0.23	5.00	23.70	36.97	59.50	0.31	0.07				
	12	0.63	0.58	4.94	7.18	12.41	6.25	0.08	0.04	0.078	0.062	0.012	-1.376
	24	0.85	0.78	4.51	8.82	11.09	8.50	0.10	0.07	0.024	0.026	0.048	0.137
	36	1.22	1.12	4.40	8.62	0.14	12.25	0.09	0.11	0.031	0.030	0.009	-0.017
	48	1.75	1.58	3.98	9.98	0.09	17.50	0.11	0.17	0.028	0.030	0.024	0.114
	60	2.43	2.12	3.78	12.38	0.03	24.25	0.14	0.30	0.025	0.027	0.008	0.200
	72	3.17	2.73	3.13	14.16	0.00	31.75	0.17	0.45	0.021	0.022	0.019	0.148
	84	3.57	3.17	2.66	11.34	0.01	35.75	0.13	0.41	0.013	0.010	0.012	-0.236
	96	3.33	2.97	2.21	10.65	0.01	33.25	0.12	0.35			0.011	-0.057
Alanine	0	0.30	0.23	5.00	23.70	36.97	59.50	0.31	0.07				
	12	0.17	0.15	4.62	11.67	63.36	1.75	0.13	0.02			0.135	-1.003
	24	0.77	0.67	4.44	13.61	8.95	7.75	0.16	0.11	0.122	0.124	0.032	0.162
	36	1.40	1.18	3.44	15.79	5.33	14.00	0.19	0.22	0.047	0.049	0.076	0.182

	48	1.78	1.47	2.67	16.90	3.41	17.75	0.20	0.30	0.019	0.020	0.040	0.092
	60	1.95	1.60	1.35	17.97	4.77	19.50	0.22	0.35	0.007	0.008	0.059	0.089
	72	2.35	1.94	1.19	17.65	2.89	23.50	0.21	0.41	0.016	0.016	0.006	-0.027
	84	2.78	2.25	1.07	18.93	3.38	27.75	0.23	0.53	0.013	0.014	0.004	0.106
	96	2.98	2.47	0.76	16.81	2.14	29.75	0.20	0.50	0.008	0.006	0.009	-0.176
Glutamate	0	0.30	0.23	5.00	23.70	36.97	59.50	0.31	0.07				
	12	0.72	0.64	4.91	11.71	0.00	7.25	0.13	0.08	0.086	0.074	0.014	-0.999
	24	0.98	0.84	4.75	13.84	0.00	9.75	0.16	0.13	0.023	0.025	0.016	0.177
	36	1.45	1.23	4.24	15.19	4.07	14.50	0.18	0.22	0.032	0.033	0.035	0.112
	48	2.15	1.79	3.73	16.94	66.53	21.50	0.20	0.36	0.031	0.033	0.024	0.146
	60	3.00	2.47	2.67	17.51	7.88	30.00	0.21	0.53	0.027	0.028	0.034	0.047
	72	3.57	2.83	1.66	20.98	3.22	35.75	0.27	0.75	0.011	0.015	0.026	0.289
	84	3.78	3.07	0.75	18.55	26.02	37.75	0.23	0.70	0.007	0.005	0.021	-0.203
	96	3.90	3.23	0.44	17.06	25.32	39.00	0.21	0.67	0.004	0.003	0.007	-0.124
Aspartate	0	0.30	0.23	5.00	23.70	36.97	59.50	0.31	0.07				
	12	0.22	0.21	4.62	6.50	96.93	2.25	0.07	0.01			0.122	-1.433
	24	0.52	0.49	4.37	7.55	5.86	5.25	0.08	0.04	0.070	0.071	0.054	0.087
	36	0.70	0.63	3.80	9.29	0.48	7.00	0.10	0.07	0.022	0.024	0.078	0.145
	48	1.05	0.94	3.83	10.05	0.19	10.50	0.11	0.11	0.033	0.034	-0.003	0.063
	60	1.27	1.18	3.50	7.56	0.19	12.75	0.08	0.10	0.018	0.016	0.024	-0.207
	72	1.70	1.59	2.91	6.74	0.65	17.00	0.07	0.11	0.025	0.024	0.033	-0.068
	84	1.90	1.78	1.41	6.33	0.92	19.00	0.07	0.12	0.010	0.009	0.070	-0.034
	96	2.18	2.05	1.39	5.75	0.45	21.75	0.06	0.13	0.012	0.011	0.001	-0.049
Serine	0	0.32	0.25	5.00	21.42	2.34	63.50	0.27	0.07				
	12	0.20	0.17	4.66	12.67	31.05	2.00	0.15	0.03			0.108	-0.729
	24	0.35	0.29	4.26	15.83	8.96	3.50	0.19	0.06	0.044	0.047	0.123	0.264
	36	0.80	0.67	3.93	16.82	2.92	8.00	0.20	0.13	0.068	0.069	0.047	0.083
	48	1.15	0.94	3.09	18.26	0.00	11.50	0.22	0.21	0.029	0.030	0.072	0.120

	60	1.63	1.28	2.46	21.23	0.03	16.25	0.27	0.35	0.026	0.029	0.038	0.248
	72	2.07	1.67	2.32	19.28	3.42	20.75	0.24	0.40	0.022	0.020	0.006	-0.163
	84	2.67	2.28	1.60	14.62	0.71	26.75	0.17	0.39	0.026	0.021	0.026	-0.389
	96	3.63	3.09	0.97	14.88	0.17	36.25	0.17	0.54	0.025	0.025	0.017	0.022
Leucine	0	0.32	0.25	5.00	21.42	2.34	63.50	0.27	0.07				
	12	0.40	0.38	4.78	5.08	24.09	4.00	0.05	0.02	0.035	0.019	0.052	-1.361
	24	0.80	0.75	4.60	5.63	6.52	8.00	0.06	0.05	0.057	0.058	0.025	0.045
	36	1.05	0.97	4.04	7.78	3.09	10.50	0.08	0.08	0.021	0.023	0.050	0.179
	48	1.65	1.47	3.68	10.89	1.65	16.50	0.12	0.18	0.035	0.038	0.023	0.259
	60	1.93	1.67	2.71	13.25	1.41	19.25	0.15	0.26	0.011	0.013	0.045	0.197
	72	2.47	2.20	2.05	11.09	1.19	24.75	0.12	0.27	0.023	0.021	0.025	-0.180
	84	3.03	2.73	0.05	9.60	0.89	30.25	0.11	0.29	0.018	0.017	0.061	-0.124
	96	3.75	3.37	0.00	10.26	0.76	37.50	0.11	0.38	0.017	0.018	0.001	0.055
Pheylalanine	0	0.32	0.25	5.00	21.42	2.34	63.50	0.27	0.07				
	12	1.05	0.99	4.91	5.83	0.26	10.50	0.06	0.06	0.115	0.100	0.012	-1.299
	24	1.63	1.49	4.37	8.61	2.64	16.25	0.09	0.14	0.034	0.036	0.034	0.231
	36	1.93	1.73	3.61	10.12	1.45	19.25	0.11	0.19	0.013	0.014	0.036	0.126
	48	2.32	2.03	3.53	12.49	0.00	23.25	0.14	0.29	0.014	0.016	0.003	0.197
	60	2.63	2.21	2.76	15.62	10.11	26.25	0.19	0.41	0.007	0.010	0.026	0.262
	72	3.25	2.58	2.43	20.46	5.78	32.50	0.26	0.67	0.013	0.018	0.009	0.403
	84	3.40	2.81	1.32	17.35	4.14	34.00	0.21	0.59	0.007	0.004	0.028	-0.259
	96	3.72	3.17	0.75	14.77	3.57	37.25	0.17	0.55	0.010	0.008	0.013	-0.215
Untreated	0	0.31	0.24	5.00	22.56	14.68	61.83	0.29	0.07				
inoculum	12	1.85	1.61	4.22	12.97	12.14	18.50	0.15	0.24	0.159	0.149	0.070	-0.799
	24	3.55	3.03	3.49	14.64	5.22	35.50	0.17	0.52	0.053	0.054	0.026	0.140
	36	3.95	3.28	1.39	16.86	5.32	39.50	0.20	0.67	0.007	0.009	0.055	0.184
	48	4.08	3.22	0.12	21.00	3.84	40.75	0.27	0.86	-0.002	0.003	0.033	0.345
	60	4.35	3.31	0.00	23.91	0.89	43.50	0.31	1.04	0.002	0.005	0.003	0.243

	72	4.58	3.25	0.00	28.97	0.39	45.75	0.41	1.33	-0.002	0.004	0.000	0.421
	84	5.40	3.77	0.00	30.20	0.14	54.00	0.43	1.63	0.012	0.014	0.000	0.103
	96	6.03	4.41	0.00	26.86	0.08	60.25	0.037	1.62	0.013	0.009	0.000	-0.278
Yeast extract	0	0.31	0.24	5.00	22.56	14.68	61.83	0.29	0.07				
1 g/L	12	0.28	0.25	4.34	9.00	0.00	2.75	0.10	0.02	0.004	-0.10	0.223	-1.130
	24	0.55	0.48	3.70	12.73	0.20	5.50	0.15	0.07	0.054	0.058	0.148	0.311
	36	1.12	0.97	2.05	13.75	0.08	11.25	0.16	0.15	0.059	0.060	0.189	0.085
	48	1.75	1.46	1.48	16.60	10.28	17.50	0.20	0.29	0.034	0.037	0.039	0.237
	60	2.60	2.13	0.87	17.91	3.10	26.00	0.22	0.47	0.032	0.033	0.028	0.110
	72	2.72	2.30	0.02	15.43	1.89	27.25	0.18	0.42	0.006	0.004	0.032	-0.206
	84	3.10	2.66	0.00	14.32	1.46	31.00	0.17	0.44	0.012	0.011	0.001	-0.093
	96	3.48	3.02	0.00	13.10	1.32	34.75	0.15	0.46	0.011	0.010	0.000	-0.102
Supplem	0	0.31	0.24	5.00	22.56	14.68	61.83	0.29	0.07				
with	12	0.98	0.87	4.31	10.32	8.41	9.75	0.12	0.10	0.108	0.096	0.103	-1.020
vitamins	24	1.40	1.20	3.78	14.59	0.20	14.00	0.17	0.20	0.026	0.030	0.043	0.356
	36	3.30	2.71	1.98	18.01	0.41	33.00	0.22	0.59	0.068	0.071	0.077	0.285
	48	3.83	3.05	1.41	20.13	1.08	38.25	0.25	0.77	0.010	0.012	0.016	0.177
	60	4.50	3.47	0.03	22.88	0.05	45.00	0.30	1.03	0.011	0.014	0.035	0.229
	72	5.35	4.06	0.00	24.13	0.06	53.50	0.32	1.29	0.013	0.014	0.001	0.104
	84	5.78	4.22	0.00	26.93	0.06	57.75	0.37	1.56	0.003	0.006	0.000	0.233
	96	5.60	4.22	0.00	24.65	0.07	56.00	0.33	1.38	0.000	-0.003	0.000	-0.190
Control	0	0.31	0.24	5.00	22.56	14.68	61.83	0.29	0.07				
	12	1.27	1.12	4.40	22.56	14.68	61.83	0.25	0.29	0.129	0.118	0.073	0.000
	24	2.42	2.07	3.85	11.25	7.97	12.67	0.13	0.27	0.051	0.054	0.029	-0.943
	36	2.98	2.47	3.38	14.50	3.07	24.17	0.18	0.43	0.015	0.018	0.017	0.271
	48	3.32	2.68	1.83	17.19	3.16	29.83	0.21	0.57	0.007	0.009	0.050	0.225
	60	3.83	2.96	0.37	19.29	0.26	33.17	0.25	0.74	0.008	0.012	0.043	0.175
	72	4.52	3.32	0.00	22.73	0.85	38.33	0.31	1.03	0.010	0.014	0.010	0.286

84	4.97	3.74	0.00	26.49	0.71	45.17	0.35	1.32	0.010	0.008	0.000	0.314
96	5.51	4.29	0.00	24.74	0.77	49.67	0.32	1.36	0.011	0.009	0.000	-0.146

Specific growth rate of total biomass: $\mu_{xt} = \frac{1}{12} Ln\left(\frac{xt_{i+1}}{xt_i}\right)$ (S.1)

Specific growth rate of the lipid free-biomass: $\mu_{xlf} = \frac{1}{12} Ln\left(\frac{xlf_{i+1}}{xlf_i}\right)$ (S.2)

Specific rate of glucose consumption: $r_g = \frac{-1}{av(xt_i, xt_{i+1})} \frac{1}{12} (g_{i+1} - g_i)$ (S.3)

Specific rate of total lipids synthesis/consumption: $r_L = \frac{1}{av(xt_i, xt_{i+1})} \frac{1}{12} (L_{i+1} - L_i)$ (S.4)

Table S2. Composition of the vitamins and minerals of the YNB (control) medium and YNB medium supplemented with vitamins and trace mineral solutions used to grow *Thraustochytrium* sp. RT2316-16.

**YNB-control (mg/L)**		**YNB**	**YNB+supp.**
**Vitamins**		mg L^{-1}	mg L^{-1}
Biotin		0.002	0.038
Calcium pantothenate		0.400	0.472
Folic acid		0.002	0.002
Niacin		0.4	0.472
p-Aminobenzoic acid		0.2	0.272
Pyridoxine HCl		0.4	0.429
Riboflavin		0.2	0.56
Thiamine HCl		0.4	0.544
Inositol		2	2
Cobalamin		0	0.004
Pyridoxamine		0	0.72
**Inorganic**			
Boric acid	H3BO3	0.5	0.5
Copper sulfate	CuSO4	0.04	4.84
Potassium iodide	KI	0.1	0.1
Ferric chloride	FeCl3	0.2	0.2
Manganese sulfate	MNSO4	0.4	0.4
Sodium molybdate	Na2MoO4	0.2	0.2
Zinc sulfate	ZnSO4	0.4	7.6
Manganese chloride tetrahydrate	MnCl2.4H2O	0	7.2
Cobalt chloride hexahydrate	CoCl2.6H2O	0	0.096
Ferrous sulfate heptahydrate	FeSO4.7H2O	0	24
Nickel sulfate hexahydrate	NiSO4.6H2O	0	4.8
Potassium phosphate monobasic	KH2PO4	1000	1120
Magnesium sulfate	MgSO4	500	500
Sodium chloride	NaCl	100	100
Calcium chloride	CaCl2	100	100

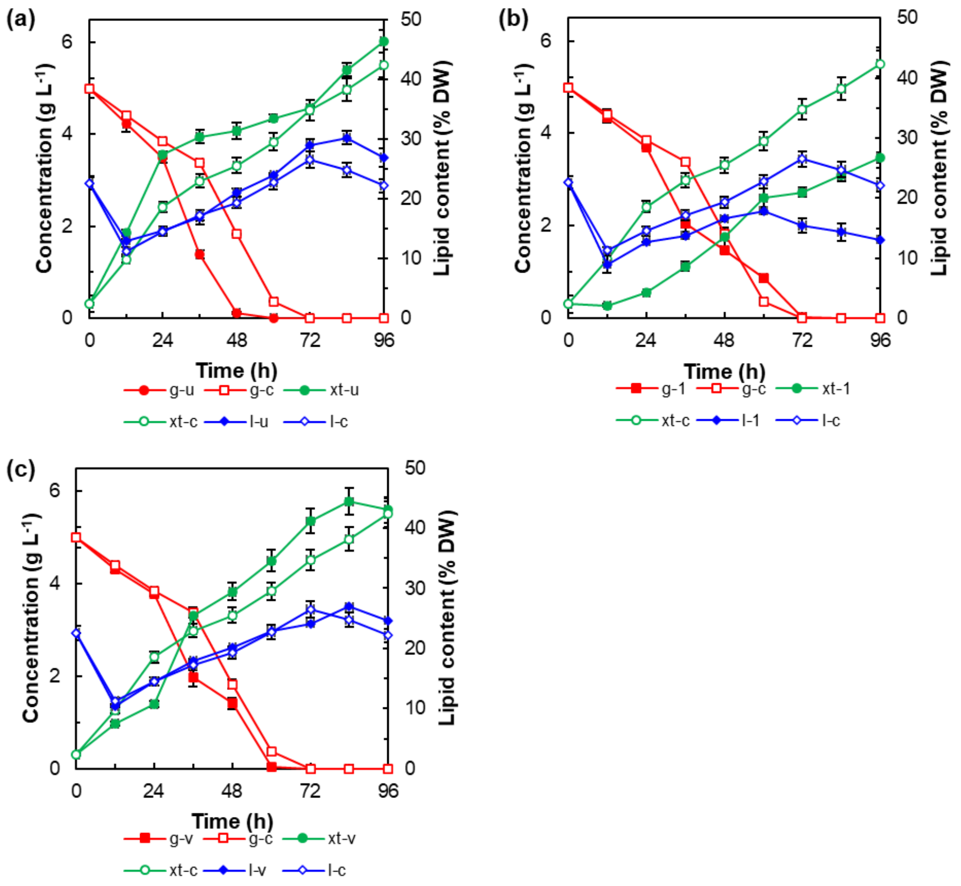

Figure S1. Effect of: (a) the inoculum (c, aliquot of washed cells; u, aliquot of non-washed cells), (b) yeast extract concentration (1 and 6 (c) g L^{-1}), and (c) extra vitamins and trace minerals (v) on the evolution of the concentration of total biomass (xt), the residual glucose (g) and the content of total lipids (l) in the total biomass of *Thraustochytrium* sp. RT2316-16 during the incubation (15 °C, 150 rpm).

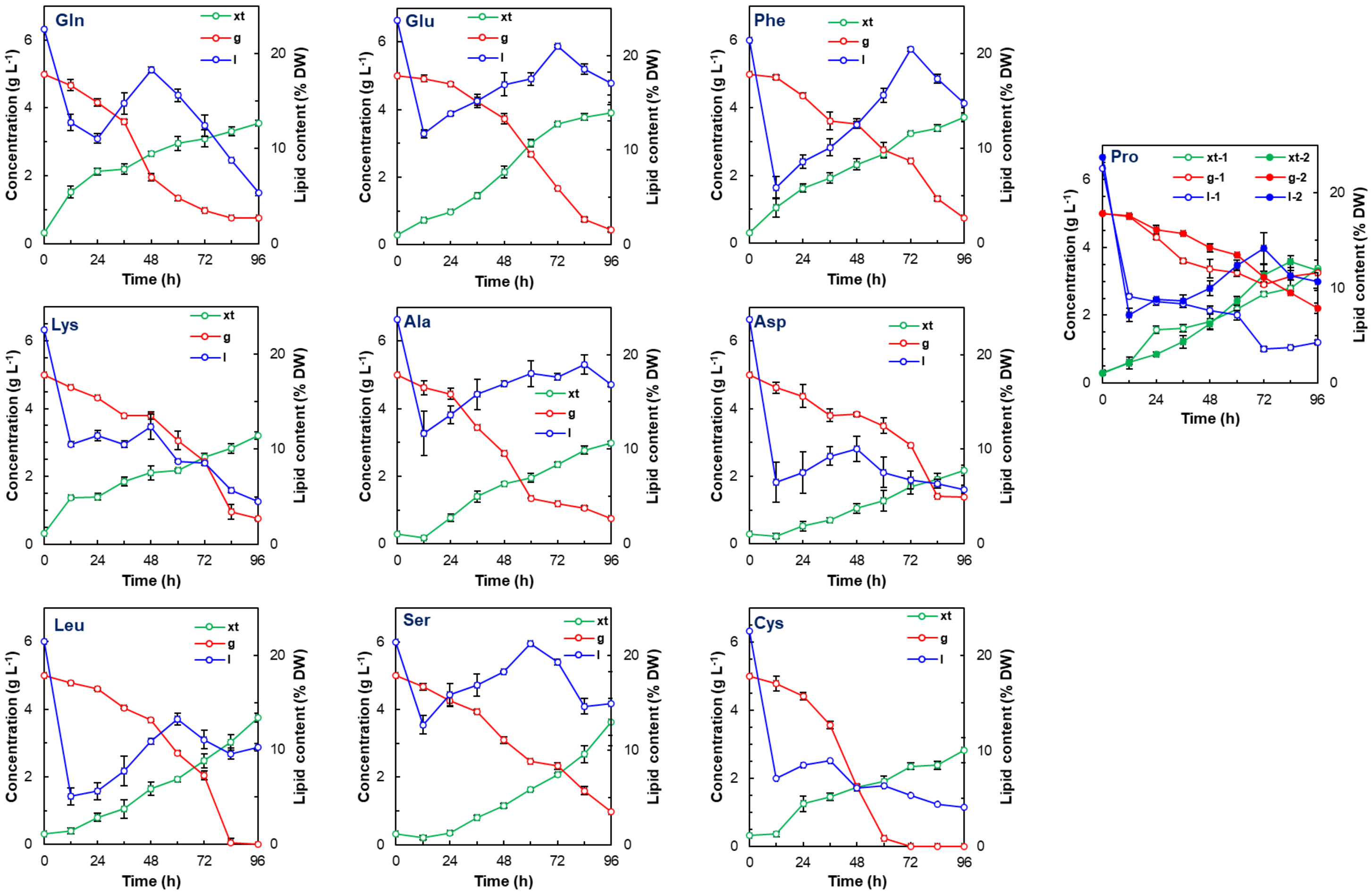

Figure S2. Effect of the amino acid used as the sole nitrogen source for growing *Thraustochytrium* sp. RT2316-16 on the evolution of the concentration of total biomass (xt), and residual glucose (g), and the total lipid content (l) of the biomass. Incubation was madea at 15°C, and 150 rpm. The amino acids tested were glutamine, Gln; glutamate, Glu; phenylalanine, Phe; proline, Pro (at 1 and 2 g L^{-1}); lysine, Lys; alanine, Ala; aspartate, Asp; leucine, Leu; serine, Ser; and cysteine, Cys.
